# The magnitude and determinants of antepartum depression among women attending antenatal clinic at a tertiary hospital, in Mwanza Tanzania: a cross-sectional study

**DOI:** 10.11604/pamj.2021.38.258.27023

**Published:** 2021-03-12

**Authors:** Matiko Mwita, Doreen Kasongi, Eliya Bernard, Daniel Gunda, Blandina Mmbaga

**Affiliations:** 1 Catholic University of Health and Allied Sciences, Mwanza, Tanzania,; 2 Bugando Medical Centre, Psychiatry Department, Mwanza, Tanzania,; 3 Kilimanjaro Christian Medical University College, Moshi, Tanzania

**Keywords:** Pregnancy, mental health, depression, women, Tanzania, antenatal

## Abstract

**Introduction:**

there is an increased vulnerability for the development of common mental disorders during the peripartum period as evidenced in depressive disorder.

**Methods:**

a cross sectional study was used to determine the prevalence and risk factors associated with depression among pregnant women attending antenatal clinic (ANC) at Bugando Medical Centre (BMC), a tertiary level hospital in Mwanza Tanzania. A total of 380 pregnant women were recruited and interviewed by using Edinburg Postnatal Depression Scale. The sample size was randomly selected from the clinic.

**Results:**

the mean age of the participants was 30.35 years, with minimum and maximum age of 20 years and 47 years respectively with 89.74% of the participants being married. Almost half of the participants, 53.68% were on the third trimester, with about two-third of the participants, 76.84% reports to have planned for their current pregnancies. The overall prevalence of depression was 15% with middle age of the partner (31-40 years), been married, high level of education, partner and family support were found to be statistically protective for depression while polygamy and partner violence were statistically risk factor for depression.

**Conclusion:**

the results showed high prevalence of antepartum depression which emphasizes the importance of earlier screening, detection and intervention to reduce the burden of morbidity and disability.

## Introduction

There is an increased vulnerability for the development of common mental disorders among perinatal and postnatal women, including depression [1]. Antepartum depression is a nonpsychotic depressive episode of mild to moderate severity that presents during pregnancy [2]. Perinatal common mental disorders (PCMDs) such as depression are three times more prevalent in low-and middle-income countries (LMICs) than in high-income countries (HICs) [3] and a major cause of disability among women. Studies have shown that the prevalence of depression during pregnancy ranges from 7-15% in high-income countries and 19-25% in low- and middle-income countries depending on the populations studied and instruments used [3, 4] while in Tanzania, the prevalence of almost 40% for symptoms of depression among pregnant women has been reported [5].

Antepartum depression is of concern because it has been linked to negative health-related behaviors and outcomes, including poor nutrition, increased substance use, inadequate prenatal care, miscarriages, preeclampsia, preterm delivery, low birth weight, impairments in mother-infant interactions, postnatal depression, and suicide [6, 7]. Genetic vulnerabilities, hormonal dysregulation and psychosocial factors have been postulated as risk factors for PCMDs [8]. The most significant psychosocial factors identified are severe life events, chronic strain, relationship quality, lack of partner support, partner violence, previous experience of pregnancy loss or complications, and HIV diagnosis during the index pregnancy [8-10]. The aim of this study was to determine the prevalence and factors associated with depression among pregnant women attending antenatal clinic (ANC) at Bugando Medical Centre, Mwanza Tanzania.

## Methods

**Study design and settings:** a cross-sectional study was conducted at Bugando Medical Centre a tertiary referral, teaching and research Centre for the Lake and Western zones of the United Republic of Tanzania. The hospital has 1000 beds and serves a catchment population of approximately 15 million people, about 500 women attends ANC at BMC per month [11].

**Sample size, participants´ enrolment and data collection:** the study population involved all pregnant women attending antenatal clinic at Bugando Medical Centre. A minimum sample size of 345 participants was estimated from Kish-Lisle formula of cross-sectional studies, assuming about 33.8% of all ANC women will have depression [12]. All pregnant women who presented for ANC at BMC during the study period were invited to participate. The aim of the study was explained and consent to participate was requested. After signing an informed consent, participants were asked to complete self-administered research questionnaires starting with the socio demographic followed by Edinburg Postnatal Depression Scale (EDPS), the data entry was done using an Epi info software. Participants were serially enrolled until the sample size was reached. Physically ill participants were excluded from the study. Those scored moderate or high on EDPS or have active suicidal behavior were referred and escorted to the psychiatry clinic found within the hospital for further clinical evaluation and intervention.

**Data analysis:** data was analyzed using Stata version 13 software for Windows where categorical variables were summarized using frequencies and percentages and continuous variables were summarized using medians with interquartile range (IQR). Descriptive analysis was conducted to describe the socio-demographic characteristics, the prevalence and severity of depression which was primary outcome in this study and participants were regarded to have depression if scored above 4 on the Edinburg Postnatal Depression Scale (EDPS) [13]. Logistic regression was used to calculate the odds ratio and 95%CI to assess the association between different factors and the outcome of interest while controlling for possible confounders. Variables in the univariate analysis were considered for inclusion into the final multivariable logistic model if they had a p<0.2 and the level of significance in the final model was set at p<0.05.

**Ethics:** ethics approval to conduct and publish the findings from this study was given from Catholic University of health and Allied Sciences/Bugando Medical Centre Joint Ethical Committee with an ethical clearance certificate number CREC/406/2019 and a further permission to conduct this study was granted by BMC administrations. Patients identifies were not used in analysis to further maintain confidentiality.

## Results

**Socio demographic characteristics:** the mean age of the participants was 30.35 years, with minimum and maximum ages of 20 years and 47 years respectively. The mean age of the participant´s partners was 35.68 years with minimum and maximum ages of 20 years and 60 years respectively. About half of the participants, 51.84% (n=197) were in the age group of 20-30 years and more than two quarter of the participants, 89.74% (n=341) were married, with monogamy being the most common type of marriage, 97.63% (n=371). More than half of the participant´s partners, 58.95% (n=225) were at the age group of 31-40 years. Almost half of the participants, 53.68% (n=204) were on the third trimester, with about two-third of the participants, 76.84% (n=292) reports to have planned for their current pregnancies. Majority of the participants, 95.53% (n=363) reported partner support and 96.58% (n=367) reported family support. Table 1 summarizes the socio-demographic characteristics of the study participants.

**Table 1:** socio-demographic characteristics of pregnant women attending antenatal clinic at Bugando Medical Centre

Variable	Frequency (n)	Percentage (%)
**Age of mother (years)**		
20-30	197	51.84
31-40	172	45.26
>40	11	2.89
**Age of partner (years)**		
20-30	81	21.32
31-40	225	58.95
41-50	70	18.42
>50	5	1.32
**Marital status**		
Never married	39	10.26
Married	341	89.74
**Type of marriage**		
Monogamy	371	97.63
Polygamy	9	2.37
**Education level**		
Never go to school	5	1.32
Primary	57	15.00
Secondary	114	30.00
College	92	24.21
University	112	29.47
**Occupation**		
House wife	59	15.53
Self-employment	120	31.58
Employed	201	52.89
**Income (monthly) TShs**		
<100,000	92	24.21
100001-300000	73	19.21
300001-500000	104	27.37
500001-999999	78	20.53
>999999	33	8.68
**Gravidity**		
Prime	83	21.84
Multiparous	297	78.16
**Trimester**		
1^st^	40	10.53
2^nd^	136	35.79
3^rd^	204	53.68
**Planned-for the pregnancy**		
No	88	23.16
Yes	292	76.84
**Previous pregnancy loss**		
No	236	62.11
Yes	144	37.89
**Previous baby loss**		
No	337	88.68
Yes	43	11.32
**Partner support**		
No	17	4.47
Yes	363	95.53
**Family support**		
No	13	3.42
Yes	367	96.58
**Partner violence**		
No	362	95.26
Yes	18	4.74
**Smoking history**		
No	372	97.89
Yes	8	2.11
**HIV status**		
Negative	350	92.11
Positive	30	7.89

**Prevalence of depression among pregnant women attending ANC at BMC:** prevalence and severity of depression were classified using scores derived from the Edinburg Postnatal Depression Scale (EDPS). Out of the possible maximum score of 30, the 380 study participants had an average score of 4.90. The lowest score recorded in the sample was 0 and the highest score was 23. The prevalence of depression was found to be 15%. The remaining 85.00% of participants did not have depression (EPDS score from 0-10).

**Factors associated with depression:** in an unadjusted model, age of the partner, type of marriage, level of education, employment, partner support, family support and partner violence were significantly associated with depression. After adjusting for other covariates, women whose partners were at the age of 31-40 years were statistically less likely to develop depressive symptoms (adjusted odds ratio (AOR) 0.4, 95% CI: 0.2, 0.8, p=0.020) the same applies to those who were married (AOR 0.4, 95% CI: 0.2, 0.8, p=0.026). Type of marriage was statistically associated with depression with those in polygamy type of marriage being more likely to develop depressive symptoms (AOR 9.8, 95% CI: 2.1, 45.2, p= 0.003). Those attained college level of education were less likely to develop depressive symptoms (AOR 0.1, 95% CI: 0.0, 0.8, P-value 0.030) compared to those with no formal education. Those with partner support and family support were both significantly less likely to develop depressive symptoms with (AOR 0.1, 95% CI: 0.1, 0.6, p=0.008) and (AOR 0.2, 95% CI: 0.1, 0.9, p= 0.041) respectively, compared to those reported neither partner nor family support. While on the other hand those who reported partner violence were statistically more likely to develop depressive symptoms (AOR 7.1, 95% CI: 2.1, 23.4, p=0.001) compared to those did not report partner violence. Table 2 summarizes the association between sociodemographic factors and depression among study participants.

**Table 2:** association between socio-demographic factors and depression among pregnant women attending antenatal clinic at Bugando Medical Centre

Variable		Depression		Unadjusted OR (95% CI)		Adjusted OR (95% CI)	
		Yes (N %)	No (N %)	OR (95CI)	P value	OR(95%CI)	P value
**Age mother (years)**							
	20-30	32 (16.24)	165 (83.76)	1.0		1.0	
	31-40	23 (13.37)	149 (86.63)	0.7 (0.4-1.4)	0.440	0.9 (0.4-1.6)	0.759
	>40	2 (18.18)	9 (81.82)	1.1 (0.2-5.6)	0.866	1.4 (0.2-7.0)	0.657
**Age partner (years)**							
20-30		18 (22.22)	63 (77.78)	1.0			
31-40		25 (11.16)	199 (88.84)	0.4 (0.2-0.9)	0.016	0.4 (0.2-0.8)	0.020
41-50		12 (17.14)	58 (82.86)	0.7 (0.3-1.6)	0.436	0.7 (0.3-1.7)	0.520
>50		2 (40.00)	3 (60.00)	2.3 (0.3-15.1)	0.373	1.9 (0.2-13.4)	0.497
**Marital status**							
	Not married	10 (25.64)	29 (74.36)	1.0		1.0	
	Married	47 (13.78)	294 (86.22)	0.4 (0.2-1.1)	0.054	0.4 (0.2-0.8)	0.026
**Type of marriage**							
Monogamy		51 (13.75)	320 (86.25)	1.0		1.0	
Polygamy		6 (66.67)	3 (33.33)	12.5 (3.0-51.76)	0.000	9.8 (2.1-45.2)	0.003
**Education level**							
Never go to school		2(40.00)	3(60.00)	1.0		1.0	
Primary		11 (19.30)	46 (80.70)	0.4 (0.1-2.4)	0.292	0.3 (0.0-2.1)	0.239
Secondary		20 (17.54)	94 (82.46)	0.3 (0.1-2.0)	0.227	0.2 (0.0-1.8)	0.197
College		7 (7.61)	85 (92.39)	0.1 (0.0-0.8)	0.035	0.1 (0.0-0.8)	0.030
University		17 (15.18)	95 (84.82)	0.2 (0.0-1.7)	0.166	0.2 (0.0-1.5)	0.131
**Employment**							
House wife		14 (23.73)	45 (76.27)	1.0		1.0	
Self-employment		19 (15.83)	101 (84.17)	0.6 (0.2-1.3)	0.203	1.6 (0.3-1.4)	0.241
Employed		24 (11.94)	177 (88.06)	0.4 (0.2-0.9)	0.027	0.4 (0.2-1.1)	0.058
**Planned-for the pregnancy**							
No		14 (15.91)	74 (84.09)	1.0		1.0	
Yes		43 (14.73)	249 (85.27)	0.9 (0.5-1.8)	0.785	0.9 (0.5-1.7)	0.774
**Partner support**							
No		9 (52.94)	8 (47.06)	1.0		1.0	
Yes		48 (13.22)	315 (86.78)	0.1 (0.0-0.4)	0.000	0.1 (0.1-0.6)	0.008
**Family support**							
No		5 (38.46)	8 (61.54)	1.0		1.0	
Yes		52 (14.17)	315 (85.83)	0.2 (0.1-0.8)	0.024	0.2 (0.1-0.9)	0.041
**Partner violence**							
No		46 (12.71)	316 (87.29)	1.0		1.0	
Yes		11 (61.11)	7 (38.89)	10.7 (3.9-29.3)	0.000	7.1 (2.1-23.4)	0.001

## Discussion

The findings from this study suggests that depression is prevalent in almost one in seven pregnant women, same findings were observed in a systematic review study were the prevalence of 15% was reported [14] and in Bangladesh the prevalence of 18% was reported [15]. High prevalence has been reported in previous studies in high and low income countries, with the prevalence of 33% reported in USA [16], 24.3% in Brazil [17], 24.9% in Ethiopia [18] and 40% in a previous same study setting in Tanzania [5].

The different findings could be attributed by time point in pregnancy at which symptoms are assessed [19], instrument used (standard clinical interviews vs screening tools) [20], different cut-off points on screening tools used [21]. Marital status continues to play a significant role as a risk factor for antepartum depression as was observed in this study and previous studies that those who are single, divorced or separated are at higher risk of getting antepartum depression compared to those who are married [22]. Despite the fact that the married group was more protected against depression it was opposite for those in polygamous as observed in this study and other studies in sub-Saharan Africa [22, 23]. Higher level of education was found in our study to be protective against depression in this study which in line with findings from other studies from developing countries [15, 24]. This is probably due to increased social networks and social support among educated women, which has been shown to have protective effect against depressive disorders in previous studies [25-27].

The current study identified partner violence to be independently associated with depression, which is in line with a study done in rural Bangladesh where intimate partner violence, particularly physical violence was identified as contributing to both antepartum anxiety and depression [15]. The association between partner violence and perinatal common mental disorders is well documented in high-income countries [28] and growing in low-income countries [29, 30]. The findings also emphasize on the importance of partner support as evidenced in this study and previous studies [15, 31] on the prediction of women´s emotional status in the antepartum period.

**Limitations:** a cross sectional study was used which relies on self-report of symptoms which could lead to recall bias. Despite that the study was done at the largest hospital in the lake and western zone of Tanzania which serves a diverse population still regional difference could be there.

## Conclusion

There is high prevalence of antepartum depression among women attending antenatal clinic in a studied sample in Mwanza Tanzania which emphasizes the importance of earlier screening and detection of those at risk with intervention to reduce the burden of morbidity and disability.

### What is known about this topic

- The prevalence of depression of 7-15% among pregnant women have been reported in low and middle income countries;
- A high prevalence of 40% was reported in one study in a pre-urban setting in Tanzania.

### What this study adds

- A low prevalence of 15% was reported in this study from an urban setting;
- The risk of developing depression was high among women in polygamy marriage and those experiencing partner violence.
